# Interprofessional staff perspectives on the adoption of or black box technology and simulations to improve patient safety: a multi-methods survey

**DOI:** 10.1186/s41077-023-00263-2

**Published:** 2023-10-25

**Authors:** Krystle Campbell, Aimee Gardner, Daniel J. Scott, Jada Johnson, Jillian Harvey, Abby Kazley

**Affiliations:** 1 UT Southwestern Simulation Center, University of Texas Southwestern Medical Center, Dallas, TX USA; 2 Department of Healthcare Leadership and Management, College of Health Professions, Doctor of Health Administration, Medical University of South Carolina, Charleston, SC USA; 3 Department of Psychiatry, Baylor College of Medicine, Houston, TX USA; 4 Department of Surgery, University of Texas Southwestern Medical Center, Dallas, TX USA; 5 Department of Healthcare Leadership and Management, Medical University of South Carolina, Charleston, SC USA

## Abstract

**Introduction:**

Medical errors still plague healthcare. Operating Room Black Box (ORBB) and ORBB-simulation (ORBBSIM) are innovative emerging technologies which continuously capture as well as categorize intraoperative data, team information, and audio-visual files, in effort to improve objective quality measures. ORBB and ORBBSIM have an opportunity to improve patient safety, yet a paucity of implementation literature exists. Overcoming implementation barriers is critical. This study sought to obtain rich insights while identifying facilitators and barriers to adoption of ORBB and ORBBSIM in alignment with Donabedian’s model of health services and healthcare quality. Enrichment themes included translational performance improvement and real-world examples to develop sessions.

**Methods:**

Interprofessional OR staff were invited to complete two surveys assessing staff’s perceptions using TeamSTEPPS’s validated Teamwork Perceptions Questionnaire (T-TPQ) and open-ended questions. Descriptive statistics were calculated for quantitative variables, and inductive phenomenological content analysis was used for qualitative.

**Results:**

Survey 1 captured 71 responses from 334 invited (RR 21%) while survey 2 captured 47 responses from 157 (RR 29.9%). The T-TPQ score was 65.2, with Communication (70.4) the highest construct and Leadership (58.0) the lowest. Quality Improvement (QI), Patient Safety, and Objective Case Review were the most common perceived ORBB benefits. Trends suggested a reciprocal benefit of dual ORBB and ORBBSIM adoption. Trends also suggested that dual implementation can promote Psychological Safety, culture, trust, and technology comfort. The need for an implementation plan built on change management principles and a constructive culture were key findings.

**Conclusions:**

Findings supported ORBB implementation themes from previous literature and deepened our understanding through the exploration of team culture. This blueprint provides a model to help organizations adopt ORBB and ORBBSIM. Outcomes can establish an empirical paradigm for future studies.

**Supplementary Information:**

The online version contains supplementary material available at 10.1186/s41077-023-00263-2.

## Introduction

Medical errors (MEs) in hospitals contribute to over 200,000 preventable deaths annually, resulting in significant costs associated with re-admissions, longer hospital stays, and malpractice lawsuits [1–3]. Despite efforts to improve quality and develop new techniques and technologies, errors continue to plague the healthcare sector [3–5]. Surgical errors (SEs) are particularly prevalent (44.9% of MEs) and pose significant harm to patients due to the invasive nature of surgery [1, 3, 4, 6–8]. Various factors contribute to the risk of SEs, including communication breakdowns, non-adherence to safety protocols, lack of standardization, performance deviations, environmental factors, time constraints, and ineffective utilization of technology [9].

Efforts to mitigate MEs have been multi-dimensional which have included data collection, analysis, as well as dissemination at state and national levels, policy changes, and targeted initiatives [2, 9–12]. At the micro-level, efforts have focused on overcoming site-specific challenges, including performance goals, Universal Protocols, safety checklists, and in-depth systematic review of cases and errors [4, 9]. However, the outcomes of these efforts have been mixed, indicating the need for a critical evaluation of why errors persist in healthcare, concerted efforts to find new ME mitigators, and a better understanding of which mechanisms are most effective [9, 11, 12].

Growing literature on the use of audio-visual (AV) recordings in clinical environments has shown promise in improving ME identification, standardization, reporting, and mitigation through enhanced quality of objective data [13–15]. In an effort to expand the promising findings of AV recording, emerging technology has sought to enhance clinical AV recordings by harnessing big data through the capture of additional data streams [15]. Analogous to flight deck recorders, Operating Room (OR) Black Box (ORBB)(Surgical Safety Technologies Inc., Toronto, ON, Canada) continuously captures and categorizes additional sources of intraoperative data, such as patient records, physiological capture, environment data (noise, interruptions, people present), and team information alongside AV files [15]. ORBB then transforms this information into detailed reports as well as real-time benchmarks via artificial intelligence [15].

Based on ORBB's capability to enhance reporting, identify contributing factors to MEs, and improve coaching metrics, the integration of ORBB into simulation (ORBBSIM) presents a valuable opportunity to advance ME mitigation efforts. As the pioneering paper that introduces ORBBSIM, we present the following examples to provide a contextual understanding. ORBBSIM represents an innovative approach that harnesses multiple streams of data to augment simulation effectiveness, enabling the design of tailored development programs and facilitating more accurate assessments. ORBB installed systems capture data streams from a wide range of devices, such as AV data from procedural cameras (i.e., laparoscope), AV room data from ceiling-mounted cameras, simulated patient records, physiological measurements (manikin vitals), environmental factors (number of people present), and behavioral observations (i.e., teamwork, communication, or eye-tracking). By amalgamating these disparate data streams, ORBBSIM has a significant opportunity to enhance learning and patient outcomes. This personalized approach can maximize the effectiveness of simulation-based training and promote competency development in a targeted and efficient manner.

ORBB literature is expanding. The majority of literature can be categorized into the following groups. First, ORBB has demonstrated the ability to improve real-time monitoring and enhance reporting capabilities [16–25]. Next, ORBB literature has highlighted its effectiveness in capturing and organizing multiple data sets across different domains, surpassing the capabilities of legacy AV systems [16–29]. Next, studies have shown that ORBB's expanded scope in collecting multiple data streams has significantly improved the identification of contributing factors to MEs and the effectiveness of targeted mitigation efforts [16, 17, 20, 22, 25]. Additionally, the improved data capture and accuracy of ORBB have demonstrated enhanced metrics for coaching programs, outperforming the outdated apprenticeship model and accentuating the value of ORBBSIM [14, 15, 18].

Lastly, only two studies have examined ORBB implementation [26, 27]. Findings revealed a range of OR team’s attitudes towards the technology, with some expressing mixed feelings [26]. Team members who were supportive of the technology emphasized that their support was contingent upon its implementation from the perspective of promoting a patient safety culture, enhancing care processes, and improving patient outcomes [26]. This underscores the significance of adopting ORBB through an effective implementation strategy that avoids any perception of it being used for punitive purposes but rather as a learning tool [26, 27]. Stakeholders believed all staff should be included to foster buy-in while underscoring the importance of transparency [26, 27]. Those who were opposed to ORBB cited concerns about feeling threatened, fear of a punitive culture, downstream legal challenges, and the breach of confidentiality [26]. Findings corroborate with clinical recording implementation literature, where stakeholders believed recordings could improve patient care, but only with careful implementation [26, 27, 30–36]. Similarly, broader clinical recording implementation literature has found those opposed to this technology cited concerns about feeling threatened, a punitive culture, legal challenges, and privacy [26, 37]. These single-site studies focused on clinical adoption with no known literature on ORBB adoption in simulation [26, 34–36].

**Fig. 1:**
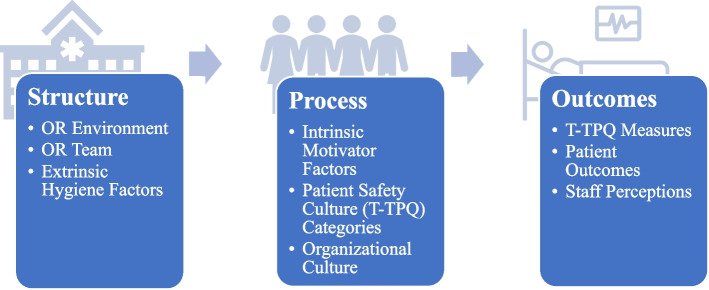
Donabedian’s quality framework. Figure 1 outlines the application of Donabedian’s quality framework to the project, highlighting conceptually the relationships between different quality improvement factors

To expand the scope and maximize the impact of ORBB and ORBBSIM across the industry, effective adoption across all disciplines is critical [21, 26–30]. Endemically, healthcare has struggled with poor adoption of technology, as seen in the cases of pre-COVID telehealth and system-wide simulation [31, 32]. Implementation inhibitors encompass complex infrastructures (structure), diverse stakeholder perspectives (process), and a legacy punitive culture (outcomes) (Fig. 1) [31–33, 37–39]. Individual-level barriers include ignorance, poor communication, and apathy, while system-level barriers encompass a lack of critical information, implementation resources, and feedback mechanisms [35, 39–44] . An implementation plan that harnesses a system’s approach, like Donabedian’s Model, can ensure all factors that impact adoption are examined and considered [21, 26–30, 40–44].

### Aim

To address these gaps, this study sought to obtain rich insights while identifying facilitators and barriers to the adoption of ORBB and ORBBSIM, aligning with Donabedian's model of health services and healthcare quality (Fig. 1). Donabedian’s *Process* factors were measured via AHRQ’s Team Strategies and Tools to Enhance Performance and Patient Safety (TeamSTEPPS) Teamwork Perceptions Questionnaire (T-TPQ) [45–47]. *Outcomes* were measured through qualitative questions exploring staff perspectives [42, 48]. Findings will enable teams to better adopt ORBB technology in order to enhance effective error mitigation leading to improved patient outcomes. The study employs a multi-methods approach to provide critical descriptive data for the effective adoption of ORBB and ORBBSIM.

## Methodology

This was a prospective convergent multi-methods study using descriptive and qualitative data solicited via electronic surveys [47, 49]. A multi-method approach enabled a rich understanding of underlying staff perceptions, beliefs through lived experiences, and culture through methodological triangulation [49]. Surveys provided a method to capture a diverse range of perspectives from a larger audience in an efficient manner while ensuring confidentiality and encouraged non-biased responses, thus promoting honest and representative data collection [49]. The study was designed based on the Standards for Reporting Qualitative Research checklist [50]. The UTSW Institutional Review Board approved an exemption based on Human Research Subject Regulations.

Two subsequential surveys were administered to interprofessional OR team members working at UT Southwestern (UTSW) Medical Center’s Clements University Hospital (CUH). CUH is a large 751-bed academic hospital and was selected because it recently installed ORBBs in five out of 59 (8.5%) ORs; all five ORBBs were in robotic ORs. Additionally, UTSW is the first US center to install ORBBs in simulated ORs. Leadership’s buy-in was garnered through the ORBB Executive Committee. UTSW policies around sensitive data required the first survey (S1) to be administered through the organizational survey system, GLINT (Sunnyvale, CA). Open-ended qualitative questions were administered via a subsequent secondary survey (S2) through REDCap electronic data capture tools.

### Survey development

S1’s quantitative questions captured staff’s attitudes towards five core T-TPQ team culture constructs [47, 49, 51, 52]. T-TPQ examined micro- and macro-level factors via five core components’ subscales, including Team Structure, Leadership, Mutual Support, Situation Monitoring, and Communication [47]. Seven Likert-scale questions defined and later coded each of the five subscales, where 100 = strongly agree, 50 = neutral, and 0 = strongly disagree (see Supplemental Digital Content 1 for a copy of the survey 1) [47]. S2 contained qualitative questions which elicited naturally occurring phenomena around interprofessional teams’ perspectives, beliefs, and feelings about ORBB, ORBBSIM, perceived benefits, and barriers (Supplemental Digital Content 2 for a copy of the survey 2) [40, 42, 47, 49, 51]. Five subject matter experts reviewed surveys’ instrument wording, flow, design, and collection format [49].

### Survey administration

S1 was administered to the full population of 334 CUH OR-members working in robotic ORs. The email invited the following team members (% of 334 invited) to the survey: medical doctors (surgeons and anesthesiologists) (32%), nurses (35%), technicians (13%), APPs (19%), and other OR staff (1%) to the survey. S1 was open from November 29, 2021, through December 21, 2021, and took an average of 30–40 min to complete. S2 was administered to a subgroup of S1, focusing on a core group of 157 ORBB interprofessional team members who were identified by CUH OR leadership as those who work primarily in rooms outfitted by ORBB. This included medical doctors (surgeons and anesthesiologists) (39%), nurses (31%), technicians (9%), APPs (20%) and other OR staff (1%) to the survey. S2 was administered from December 22, 2021, through January 22, 2022, and took an average of 10–20 min to complete.

Participants for both surveys were invited via email to participate. The email included an introduction, the aim, completion time, duration surveys would be open, a reminder that the survey was voluntary, a statement ensuring invitees that answers would not impact the respondent’s job, and contact information. The email for S1 highlighted that only aggregate data would be shared. S2’s highlighted that identifiable information would only be seen by study-team members with all subsequently reported data de-identified. Weekly reminder emails were sent to those who had not completed the surveys.

### Data analysis

Descriptive statistics were computed for the quantitative variables using the validated T-TPQ survey tool's five constructs, following the defined methodology by AHRQ for calculating aggregate means. Aggregate means were determined by summing all the responses within that construct and dividing the sum by the number of items in the construct (AHRQ, 2021). To explore variations in perceptions among different healthcare professions, subgroup analysis was conducted (AHRQ, 2021). An inductive phenomenological content analysis was used to describe qualitative open-ended questions [53]. Pre-analysis, exploration, and treatment of the data were performed in Microsoft Excel (2022). The data and codes were then imported to QSr NVivo software for interpretation phases (V.11, QSR International, Doncaster, Australia). Three coders established intercoder reliability (ICR) by independently coding seven practice responses, comparing themes, and discussing areas of non-alignment. Two coders then independently coded the remaining responses and compiled a codebook. A third coder reviewed the codebook to ensure all viewpoints were considered and settled areas of non-alignment [54]. The refinement process was done by the PI using the newly developed codebook and refining codes until thematic saturation was reached, while comparing other literature’s themes [54, 55]. A summary of common emerging themes by profession using thematic content analysis was presented to coders to ensure consideration of interprofessional perspectives, mitigate personal biases, and ensure credibility [55]. Data were believed to be missing completely at random, based on variability and no observed missing data trends. Missing data were handled through listwise deletion.

## Results

### Baseline demographics

Out of the 334 surveys administered for S1, 87 responses were collected and after removing non-complete responses through listwise deletion, 76 completed responses were retained (RR 23%). Out of the 157 surveys administered for S2, 54 responses were collected and after removing non-complete responses through listwise deletion, 47 responses remained (RR 29.9%). The most common role represented for S1 was OR Registered Nurses (RN) (36, 50.7%). S2’s most common profession was Medical Doctors (MD) (30, 63.8%). S1 had an even split on respondents who had worked at UTSW for over 5 years versus under 5 years (38, 50%) and an almost even split for S2 respondents, with a slight majority working over 5 years (25, 53.2%) versus under 5 years (22, 46.8%). Looking at specific demographic S2 questions, Anesthesiology and Surgical Services had the highest and equal respondents (14, 29.8%). Respondents most commonly had simulation experience (33, 79.3%) versus no experience (14, 29.8%). The majority of respondents had worked in an OR with ORBB (37, 78.7%) and all respondents knew what ORBB was (47, 100%) (Table 1). Complete demographic information can be found in Table 1.

**Table 1:** Demographics of respondents

	**S1: T-TPQ responses (%) (** ***N*** ** = 76)**	**S2: qualitative questions (%) (** ***N*** ** = 47)**	**APPs (** ***N*** ** = 7, 14.9%)**	**MD (** ***N*** ** = 30, 63.8%)**	**OR RN (** ***N*** ** = 8, 17.0%)**	**Other (** ***N*** ** = 2, 4.2%)**
**Number of respondents by participant role (%)**						
Specialized RN-Other	5 (7.0)	1 (2.1)				
APP/Specialized RN-Anesthetist	9 (12.7)	7 (14.9)				
OR RN	36 (50.7)	8 (17.0)				
Medical Technician-OR	10 (14.1)	0 (0.0)				
Medical Doctor	6 (8.5)	30 (63.8)				
No response to question	10 (14.1)	1 (2.1)				
**Number of staff by categories of years working at UTSW (%)**						
Under 5 years	38 (50.0)	22 (46.8)				
Over 5 years	38 (50.0)	25 (53.2)				
**Survey 2 specific questions**						
**Work unit**						
Anesthesiology	NA	14 (29.8)	3 (6.4)	10 (21.3)	0 (0.0)	1 (2.1)
Many different units	NA	4 (8.5)	0 (0.0)	4 (8.5)	0 (0.0)	0 (0.0)
Pre-Op, OR/Suite, PACU/Post Op, Peri Op	NA	6 (12.8)	3 (6.4)	2 (4.3)	1 (2.1)	0 (0.0)
Surgical services	NA	14 (29.8)	0 (0.0)	8 (17.0)	5 (10.6)	1 (2.1)
Surgical unit	NA	9 (19.1)	1 (2.1)	6 (12.8)	2 (4.3)	0 (0.0)
**Number of simulations**						
1 to 2	NA	11 (23.4)	2 (4.3)	8 (17.0)	1 (2.1)	0 (0.0)
3 to 5	NA	11 (23.4)	2 (4.3)	5 (10.6)	3 (6.4)	1 (2.1)
6 to 10	NA	2 (4.3)	1 (2.1)	1 (2.1)	0 (0.0)	0 (0.0)
11 or more	NA	9 (19.1)	0 (0.0)	6 (12.8)	2 (4.3)	1 (2.1)
None	NA	14 (29.8)	2 (4.3)	10 (21.3)	2 (4.3)	0 (0.0)
**Age**						
26–35 years old	NA	9 (19.2)	2 (4.3)	4 (8.5)	2 (4.3)	1 (2.1)
36–45 years old	NA	23 (49.9)	3 (6.4)	16 (34.0)	3 (6.4)	1 (2.1)
46–55 years old	NA	12 (25.5)	1 (2.1)	9 (19.2)	2 (4.3)	0 (0.0)
56–65 years old	NA	2 (4.3)	1 (2.1)	0 (0.0)	1 (2.1)	0 (0.0)
Over 65 years old	NA	1 (2.1)	0 (0.0)	1 (2.1)	0 (0.0)	0 (0.0)
**Have worked in OR with BB**						
Yes	NA	10 (21.3)	0 (0.0)	10 (21.3)	0 (0.0)	0 (0.0)
No	NA	37 (78.7)	7 (14.9)	20 (42.6)	8 (17.0)	2 (4.2)
**Knowledge of ORBB**						
Yes	NA	47(100.0)	7 (14.9)	30 (63.8)	8 (17.0)	2 (4.2)
No	NA	0 (0.0)	0 (0.0)	0 (0.0)	0 (0.0)	0 (0.0)
**Patient Contact**						
Do NOT have direct contact w/patients	NA	1 (2.1)	0 (0)	1 (2.1)	0 (0)	0 (0)
Direct contact w/patients	NA	46 (97.9)	7 (14.9)	29 (61.7)	8 (17.0)	2 (4.2)

Highlights respondents’ demographics on both survey 1 (S1) and survey 2 (S2). S2 category of “Other” constituents one respondent not indicating their profession and one respondent who indicated a specialized nurse (non-APP/non-CRNA)

### S1: T-TPQ results

The overall T-TPQ aggregate score was 65.2. The highest AHRQ construct was Communication (70.4), followed by Situation Monitoring (68.4), Mutual Support (65.3), Team Structure (64.0), and Leadership with the lowest aggregate mean (58.0). Analyzing aggregate means by profession, Medical Technicians (MedTech) had the highest average aggregate mean (71.6) across all constructs, followed by MDs (69.7), APPs (64.1), and OR RNs (62.3), while the Other category had the lowest (53.1).

Considering constructs by profession, MedTech’s highest-rated construct was Situation Monitoring (75.4) while their lowest-rated construct was Leadership (62.3). MDs highest rated construct was Leadership (75.6), which was also the highest aggregate mean across all constructs when looking by profession. MD’s lowest construct was Team Structure (65.4). APPs highest aggregate mean was Leadership (69.0), while their lowest was Team Structure (59.7). OR RNs’ highest-scored construct was Communication (69.7), while their lowest was Leadership (46.4). Lastly, for those under the Other category, their highest score was Communication (62.1), while their lowest, also the lowest construct aggregate mean across all professions, was Leadership (42.1).

#### Team structure

Examining aggregate means for specific questions under each construct, the highest mean question under Team Structure was Responsibilities (72) while the lowest mean was Resource Efficiency (59). The profession with the highest mean rating for Team Structure was MedTechs (72.7) while the profession with the lowest mean were those assigned to the Other category (50.0).

#### Leadership

The highest mean question was Manager & Change (66.0), while the lowest mean was Manager Decision Making (52.0). The profession with the highest mean for Leadership was MDs (75.6) while the profession with the lowest mean was those assigned to Other (40.0).

#### Situation monitoring

The highest mean question under Situation Monitoring was Correct Mistakes (72.0) while the lowest mean was Staff Anticipate Needs (64.0). The group with the highest mean rating was MedTech (75.4) while the lowest were those assigned to the Other category (60.7).

#### Mutual support

The highest mean question was Caution Awareness (73.0) while the lowest mean was for Staff Conflict Resolution (54.0). The group with the highest mean under Mutual Support was MedTech (74.3) while the lowest mean was from the group Other (50.7).

#### Communication

The highest scored question under Communication was Common Terminology (77.0) while the lowest scoring question was Available Sources (64.0). The group with the highest mean was MedTechs (73.1) while the group with the lowest scores was the group, Other (62.1). Complete T-TPQ results can be found in Table 2.

**Table 2:** T-TPQ survey construct aggregate means by profession

*T-TPQ construct*	*Overall (N* = *66)*	*APPs (N* = *9, 12.7%)*	*MD (N* = *6, 8.5%)*	*Techs (N* = *10, 14.1%)*	*OR RN (N* = *36, 57.0%)*	*Other (N* = *5, 7.0%)*
*Team Structure*	64.0	59.7	65.4	**72.7**	62.4	50.0*
*Leadership*	58.0*	69.0	**75.6**	62.3	47.4	42.1*
*Situation Monitoring*	68.4	64.7	67.4	**75.4**	68.0	60.7*
*Mutual Support*	65.3	60.7	67.3	**74.3**	63.7	50.7*
*Communication*	**70.4**	66.4	72.7	**73.1**	69.7	62.1*
*Overall average mean*	65.2	64.1	69.7	**71.6**	62.3	53.1*

Values in bold text indicate the highest aggregate mean within each construct, while an asterisk (*) indicates the lowest aggregate mean. Aggregate means by profession for each question by construct are available within the supplementary materials. Ten people did not respond to the question regarding profession, thus not included within the table

### S2: Qualitative emerging themes

Across all team members, Quality Improvement (QI), Patient Safety, and Objective Case Review were the most frequently mentioned benefits for ORBB. Objective Case Review was viewed as the top benefit among APPs and MDs, with one respondent stating, “objective documentation of exactly what was and was not said as well as documentation of events.” QI and Patient Safety were the most common themes for RNs, with one respondent noting “learning from emergency situations and ability to see how we can improve especially in terms of communication” and another respondent noting, “it (ORBB) enhances patient safety measures.”

The most common concerns regarding ORBB integration were Psychological Safety (PsySaf), Privacy, and Loss of Trust. APPs and RNs most common concern was Privacy, with one respondent noting “there is a significant concern for privacy, both from the perspective of constantly being video recorded and from the perspective of the safety of recorded data in the age of constant malware attacks on healthcare institutions and their data centers”, while the most common concerns for MDs and Other was PsySaf. Complete ORBB themes based on OR team members’ perspectives can be found in Table 3.

**Table 3:** Perspectives around benefits and/or concerns with ORBB

*Themes*	Total	APPs	MDs	RNs	Other	Example statements
***OR Black Boxes (ORBB) benefit(s)***						
*Quality improvement*	**20**	2	4	**10**	**2**	“Learning from emergency situations and ability to see how we can improve. esp. in terms of communication.”
*Patient safety*	**15**	1	2	**10**	0	“It (ORBB) enhances patient safety measures.”
*Objective case review*	**13**	**3**	**5**	5	**2**	“Objective documentation of exactly what was and was not said as well as documentation of events.”
*Teamwork*	7	2	1	4	0	
*Communication*	4	0	1	4	0	
*Learning*	3	1	1	2	0	
*Process improvement*	3	0	1	1	0	
*Performance*	2	0	1	1	0	
*Compliance*	1	0	0	0	1	
*Data*	1	0	0	1	0	
*Handoffs*	1	0	0	1	0	
*Metrics*	1	0	1	0	0	
***ORBB concern(s)***						
*Psychological safety*	**21**	3	**15**	2	**1**	“It removes all trust and team building in an operating room. It is a punitive measure that discourages open communication and improvement in patient care.”
*Privacy*	**16**	**3**	9	**4**	0	“There is a significant concern for privacy, both from the perspective of constantly being video recorded and from the perspective of the safety of recorded data in the age of constant malware attacks on healthcare” institutions and their data centers
*Loss of trust*	**9**	1	8	0	0	“I’m concerned the black box data will be used for punitive purposes or to scrutinize interpersonal communication instead of being used to improve safety.”
*Data security*	5	0	4	1	0	
*Legal*	4	0	4	0	0	
*Confidentiality*	2	2	0	0	0	
*Impedes teamwork*	1	1	0	0	0	
*Stigma*	1	0	1	0	0	
*Anxiety*	1	1	0	0	0	
*Morale*	1	0	1	0	0	
*Patient safety*	1	0	1	0	0	
*Consent*	1	0	0	1	0	
*Technology*	1	0	0	0	1	

Themes are in order by frequency, with the most frequent responses listed first. Subsequently, for frequencies that appear more than once, themes are arranged alphabetically to provide a clearer presentation of the data. Bold text in row highlights the top three most common themes across all professions. Respondents’ statements provided on the most common themes

Considering ORBBSIM, the most common benefits were QI, Development of Real-world Objective Scenarios, and Education. MDs and APPs felt that QI was ORBBSIM’s top benefit. RNs viewed ORBBSIM the most beneficial to Education, noting “(ORBB) provides learning opportunities to see examples of both correct and incorrect behaviors and provide meaningful debriefing sessions after simulations". When examining team members’ concerns with ORBBSIM, the most common concerns across all professions were PsySaf, Resources, and Timing. Interestingly, PsySaf was viewed differently across the disciplines, with five respondents viewing ORBBSIM as an impediment to PsySaf. On the contrary, two respondents with different simulation experience, felt there was less concern for PsySaf when using ORBBSIM in comparison to the threat of PsySaf when ORBB is integrated clinically, with one respondent noting, “within the simulation realm the fears should be less substantial as sim sessions are already under video surveillance.” Complete ORBBSIM themes can be found in Table 4.

**Table 4:** Perspectives around benefits and/or concerns with ORBBSIM

*Themes*	Total	APP	MDs	RNS	Other	Example statements
***Benefit(s) of ORBB integration into simulation***						
***Quality improvement***	**18**	**3**	**13**	2	0	“Improved communications. Close call identification of good practice and habits for exhibition.”
***Objective scenarios***	**13**	2	10	1	0	“Gives real scenarios to increase the fidelity of the simulation.”
***Education***	**9**	0	7	**2**	0	“Provides learning opportunities to see examples of both correct and incorrect behaviors and provide meaningful debriefing sessions after simulations.”
*Metacognition*	5	1	4	0	0	
*Metrics*	4	0	4	0	0	
*Real-time data*	4	0	3	1	0	
*Teamwork*	4	1	2	1	0	
*Debriefing*	3	0	3	0	0	
*Increased fidelity*	3	0	3	0	0	
*Patient safety*	3	0	3	0	0	
*Buy in*	2	0	2	0	0	
*Communication*	2	0	1	1	0	
*Evaluation*	2	0	1	1	0	
*Psychologically safety*	2	1	1	0	0	
*Crisis resource management*	1	0	1	0	0	
*Controlled environment*	1	1	0	0	0	
*Hawthorne effect*	1	0	1	0	0	
*Low-frequency, high-acuity events*	1	0	1	0	0	
*Research*	1	1	0	0	0	
*Unclear Value Added*	1	0	1	0	0	
***Concern(s) of ORBB integration into simulation***						
***Psychological safety***	**7**	2	**5**	0	0	“Potential violation of a safe space of learning if learners are not aware of the black box recordings taking place.”
***Resources***	**6**	**3**	2	**1**	0	“Limited resources to properly simulate the complex environment in the OR.”
***Timing***	**6**	**3**	2	**1**	0	“My concern would be that it may be hard to find time for the surgical team to participate in simulation activities.”
*Privacy*	5	1	4	0	0	
*Fidelity*	2	0	2	0	0	
*Legal*	2	0	2	0	0	
*Location*	2	0	1	**1**	0	
*Cost*	1	0	1	0	0	
*Culture*	1	1	0	0	0	
*Data security*	1	0	1	0	0	
*Hawthorne effect*	1	0	1	0	0	
*Lack of inclusivity*	1	0	1	0	0	
*Lack of understanding*	1	0	1	0	0	
*Mistrust*	1	1	0	0	0	
*Schedule coordination*	1	1	0	0	0	

Themes are in order by frequency, with the most frequent responses listed first. Subsequently, for frequencies that appear more than once, themes are arranged alphabetically to provide a clearer presentation of the data. Bold text in row highlights the top three most common themes across all professions. Respondents’ statements provided on the most common themes

When asked about ORBBSIM implementation, enablers across all professions were to Engage All Stakeholders, Education/Communication, and creation of a Culture of Safety. Engagement of All Stakeholders was the most common enabler expressed by APPs, MDs, and RNs, with one respondent highlighting the importance of a, “committee with a good representation of OR members to develop accurate simulation scenarios: OR nurse, scrub tech, anesthesia tech, surgeon, CRNA/resident, anesthesiologist, medical students, and many more (radiology, vendors…).” The Other category viewed Education/Communication as the most important enabler, with one respondent noting, “will need to provide education to familiarize participants with the technology". When asked about barriers to ORBBSIM adoption, the most common barriers were Resources, Schedule, and Time. MDs and RNs were aligned that the biggest barrier was Resources, with one respondent stating, “these rooms are used for patient care and not frequently available for simulation.” APPs had a tie for their two most common barriers, split between Resources and Schedule. The Other group felt that Education and Awareness would be barriers to adoption. Lastly, when respondents were asked whether there was additional feedback on the ORBB project, the most common responses were to ensure ORBB had Inclusivity, with one respondent highlighting, “as much inclusivity across as many specialty areas as possible.” Additionally, respondents across all disciplines felt Education, Legal, Logistics, Schedule, and QI were important components of the success of ORBB and ORBBSIM. Complete ORBB and ORBBSIM implementation themes can be found in Table 5.

**Table 5:** Perspectives around ORBB integration with simulation implementation

*Themes*	Total	APPS	MDs	RNS	Other	Example statements of most common three themes
***Enablers of effective adoption of OR Black Box-enhanced simulations***						
***Engage (all) Stakeholders***	**14**	**3**	**8**	**3**	0	“Committee with a good representation of OR members to develop accurate simulation scenarios: OR nurse, scrub tech, anesthesia tech, surgeon, CRNA/resident, anesthesiologist, medical students, and many more” (radiology, vendors…)
***Education/communication***	**8**	2	5	0	**1**	“Will need to provide education to familiarize participants with the technology.”
***Culture of Safety***	**5**	0	4	1	0	“Would need to be a priority from the top. Would need to set aside specific time for the OR team to be available to do simulation activities.”
***Uphold psychological safety***	**5**	0	4	1	0	“Creating a safe space for staff to participate by having an experienced mediator or educator onsite to lead the simulations. Simulations should be comprised of team members which mimic reality, so everyone in the simulation is participating in their usual role, not playing the part of another clinician.”
*Resources*	4	1	2	1	0	
*High fidelity/realistic*	3	0	3	0	0	
*Information*	3	2	1	0	0	
*Scenario library*	2	2	0	0	0	
*Timing*	2	0	1	1	0	
*Virtual options*	2	0	2	0	0	
*Consent*	1	0	1	0	0	
*Simulation- metrics*	1	0	1	0	0	
*Simulation- policies*	1	1	0	0	0	
*Teamwork*	1	0	1	0	0	
*Trained facilitators*	1	0	1	0	0	
*Quality Improvement*	1	0	0	0	**1**	
***Barriers to the adoption of OR Black Box-enhanced simulations***						
***Resources***	**14**	**2**	**8**	**4**	0	“These rooms are used for patient care and not frequently available for simulation.”
***Schedule***	**11**	**2**	6	3	0	“Limited staffing in more areas at this time so inability to free up all members of the team.”
***Time***	**11**	1	7	3	0	“Protected time for CUH staff and nursing”
*Awareness*	5	1	2	1	**1**	
*Communication*	4	1	2	1	0	
*Cost*	4	0	2	2	0	
*Privacy*	4	0	3	1	0	
*Psychological safety*	4	0	4	0	0	
*Culture of Safety*	3	0	3	0	0	
*Data*	2	0	2	0	0	
*Education*	2	0	1	0	**1**	
*Biased QI*	1	0	1	0	0	
*Buy in*	1	0	1	0	0	
*COVID*	1	0	1	0	0	
*Expert facilitators*	1	0	1	0	0	
*Legal*	1	0	1	0	0	
*Value added*	1	1	0	0	0	

Themes are in order by frequency, with the most frequent responses listed first. Subsequently, for frequencies that appear more than once, themes are arranged alphabetically to provide a clearer presentation of the data. Bold text in row highlights the top three most common themes across all professions. Respondents’ statements provided on the most common themes

## Discussion

This is the first known study looking at staff perceptions of ORBB and ORBBSIM, and to identify interprofessional teams’ self-assessment of culture via T-TPQ (process factors) in conjunction with OR team perspectives (outcome factors) [40, 42, 45, 47].The following paragraphs highlight key areas that can promote adoption and be used as a blueprint for other organizations looking to adopt ORBB and ORBBSIM, arranged by the five AHRQ TeamSTEPPs constructs [40, 48].

Looking first at culture, the highest-scoring T-TPQ constructs were Communication, Situation Monitoring, and Mutual Support. Top-rated questions included common terminology, supporting one another to correct mistakes, and cautioning one another about dangerous situations, which highlight the existence of a supportive and collaborative team [42, 47]. Leaders can maximize these team strengths during implementation, by engaging champions early [40]. On the contrary, Leadership was found as the lowest construct when examining the overall team’s scores, but when inspecting scores by profession, MDs and APPs rated Leadership high, while MedTech, RN, and Other rated Leadership low. The dissonance between professions’ perceptions on leaders’ effectiveness demonstrates the importance of change management principles which tailor initiatives based on individuals’ or teams’ unique perspectives, beliefs, motivators, and norms (structure and process factors), promoting a leaders’ effectiveness in fostering change (outcome) [40]. The lowest-scored Leadership question of leaders not considering staff’s input when making decisions, was noteworthy when triangulated to the downstream outcomes, where a common theme was the need to engage all stakeholders, as the qualitative data help illuminate why this construct was rated lowest [40, 42, 46]. These findings further emphasize the significance of empowering diverse champions as a crucial element of effective change management. Champions play a vital role in ensuring that staff perspectives (motivators) are considered during the change process, promoting inclusivity and buy-in from all stakeholders [40, 42].

Additionally, the findings shed light on the evolving landscape of healthcare, highlighting the growing need for leaders who can foster constructive cultures. Cultivating a constructive culture creates an environment that embraces change as an opportunity for growth and improvement, enabling organizations to adapt more effectively [42]. Constructive cultures empower individuals and foster collaboration, creating a shared vision and stakeholder engagement. This collaborative approach allows organizations to navigate and adapt to change more successfully [42]. Leaders who embrace a constructive culture mindset are better equipped to execute strategic plans, generate buy-in, implement a shared vision, utilize agile change frameworks, and inspire innovation [42]. In summary, these findings not only underscore the importance of change management principles and the involvement of diverse champions in adopting ORBB and ORBBSIM, but they also highlight the essential role of visionary and adaptable healthcare leaders in establishing constructive cultures and shaping the future of healthcare [42]. It is important for leaders to use these findings as a blueprint to build infrastructures that promote constructive cultures, emphasizing the importance of seeking all stakeholders' perspectives and incorporating their input into the change process.

Next, considering Team Structure was the second lowest construct, movement towards a constructive culture would be beneficial to create a shared vision that maximizes resources, increase staff accountability, and create improved efficiency to overcome the lowest scored questions [42]. A constructive culture will also promote intrinsic and extrinsic motivators, encourage a Culture of Safety, and help teams withstand future challenges [42]. Adoption can be impeded without these focused efforts as unwanted behaviors can perpetuate a dysfunctional culture, disrupt the strategic plan execution, and inhibit innovation [42].

Similar to previous literature, all team members held common beliefs that ORBB will enhance QI, improve Patient Safety, and provide opportunities for Objective Case Review [26]. It will be important to find mechanisms that overcome concerns around the impact of ORBB on PsySaf, Privacy, and Loss of Trust (process factors). RNs seemed less concerned about trust than MDs and APPs, while instead showing more concern for privacy, similar to prior literature [26]. These findings emphasize the importance of engaging all professions and strong leadership which establishes a constructive culture in order to promote process factors of trust, PsySaf, a Culture of Safety, effective communication, as well as transparency [40, 42, 56].

All professions were aligned that ORBBSIM could enrich QI initiatives and education using objective scenarios. Enrichment themes included translational performance improvement and real-world examples to develop sessions. These are noteworthy themes, as simulation has made important advances since being highlighted as a key mechanism to improve patient safety in *To Err is Human*, but has been challenged to achieve full system-wide adoption due to a disconnect with safety system’s operations, lack of real-time data, and lack of involvement of key stakeholders [2, 32]. Based on staff’s perceptions, ORBBSIM may be a bridge that spans the chasm between the current state of simulation to system-wide adoption [32]. Respondents also recognized that ORBBSIM could increase fidelity and enhance debriefing, but stressed doing so will require the right resources, like ensuring that all teams can participate, providing protected time, considering different schedules, and using well-trained simulation experts as facilitators to preserve PsySaf. Leaders should involve stakeholders in the implementation plan to overcome these structural concerns [40]. Through triangulation, it is noteworthy that many of the lowest-scored T-TPQ questions were regarding inefficient use of resources, suggesting opportunities exist to improve resource allocation [42]. Concerns about ORBBSIM in comparison to ORBB were less about intrinsic (process) motivators (i.e., privacy or loss of trust), but more about the extrinsic (structure) barriers (i.e., resources, environment, or timing) [42]. Some respondents highlighted that simulation has established the norm of recording, thus there was less concern about ORBBSIM recordings. Indeed, simulation best practices have established safe learning environments and PsySaf as a foundational norm, through mechanisms like a Prebrief and expertly trained facilitators [57]. This suggests there might be a reciprocal benefit for ORBBSIM to also enhance the team’s PsySaf, increase ORBB comfort, and overcome privacy as well as trust concerns. Similar to other implementation studies, this study found that additional factors to expand adoption included increased education, communication, and awareness [26]. Empowering diverse champions can help establish a shared vision, strategic plan, and communication plan to enhance education, communication as well as awareness while also ensuring there is ongoing two-way communication to promote transparency [40].

### Study limitations and strengths

This is the first known single-site study to explore teams’ perceptions around ORBBSIM and the second known study exploring team’s perceptions with ORBB. Additionally, as a growing technology, though the majority of respondents knew about ORBB, a subset of respondents had yet to work clinically in a room with this technology. Thus, the degree of generalizability of our findings to all professions and other institutions is unknown. Sampling error, non-response error, varying response rates, and recall biases were limitations the study team sought to control for by ensuring correct staff solicitation, leadership buy-in, frequent follow-up, and thematic saturation [49]. This includes the fact that S2 was administered to a smaller OR population based on work location; however, the authors believe that the themes which emerged still provided rich insights regarding ORBB and ORBBSIM. Perceptions on data use might have impacted responses, but this was explicitly addressed in the survey’s accompanying email [40]. Lastly, the authors sought to methodologically triangulate the data to build a deeper understanding of adoption factors. Organizations embarking on implementation of ORBB or ORBBSIM should use this study as a blueprint for adoption strategies to first assess interprofessional staff perceptions, culture, and other factors within their own environments as future studies will be needed to understand the generalizability of findings.

### Key take-aways

Our findings supported themes from previous literature and enriched our understanding through team culture constructs, strengthening our appreciation of disruptive innovation’s impact on QI system factors [44]. As the first US organization to implement ORBBSIM, trends emerged on the reciprocal benefit of dual adoption of ORBB with ORBBSIM. These included the overwhelmingly positive beliefs that ORBB and ORBBSIM could enhance QI efforts through objective data and improve patient safety initiatives. Emerging trends also suggested that dual implementation can improve adoption through the promotion of PsySaf, team culture, trust, and technology comfort (process factors), but future studies are needed in these areas. Findings also underscored the importance of exploring a team’s culture and the adoption of a systematic implementation plan built on change management principles such that site-specific gaps (structure and process) can be overcome through involvement of all stakeholders and heightened, two-way communication [39, 41]. Lastly, our findings highlighted the importance of strong leaders who can foster constructive cultures in order to promote trust, inclusivity, and PsySaf.

## Conclusion

ORBB and ORBBSIM are promising emerging technologies to improve patient care. Our findings supported previous literature’s ORBB implementation themes, while enriching our understanding through exploration of team culture constructs. Although further research is needed, the blueprint outlined in this study provides an initial paradigm to help organizations adopt ORBB and ORBBSIM. Data and themes can be used to monitor how factors influence ORBB and ORBBSIM outcomes, providing an empirical paradigm for future studies such that consensus regarding effective implementation are achieved using best practice change management and constructive culture strategies for emerging technology in healthcare [40, 42, 43].

### Supplementary Information

**Additional file 1.**

**Additional file 2.**

## Data Availability

The datasets analyzed during the current study are not publicly available due to psychological safety and to protect sensitive data disclosed by individual team-members but are available from the corresponding author on reasonable request in aggregate form, de-identified.

## Abbreviations

APPs: Advanced Practice Providers
AV: Audio Visual
MD: Medical Doctor
ME: Medical Errors
RN: Registered Nurse
MedTech: Medical Technicians
ORBB: Operating Room Black Box
ORBBSIM: Operating Room Black Box Simulations
ORVR: Operating Room Video Recorder
PsySaf: Psychological Safety
SE: Surgical errors
SSC: Surgical Safety Checklist
S1: Survey 1
S2: Survey 2
TeamSTEPPS: Team Strategies and Tools to Enhance Performance and Patient Safety
T-TPQ: Teamwork Perceptions Questionnaire
UP: Universal Protocol
QI: Quality Improvement
