# Supplementary material for: Interprofessional staff perspectives on the adoption of or black box technology and simulations to improve patient safety: a multi-methods survey
Source: Adv Simul (Lond). 2023 Oct 25;8:24. doi: 10.1186/s41077-023-00263-2 (PMC10598903; doi:10.1186/s41077-023-00263-2)
Supplement: Supplementary file 1 — Additional file 1. [file 41077_2023_263_MOESM1_ESM.pdf]

Supplemental Digital Content 1: TeamSTEPPS Teamwork Perceptions Questionnaire

TeamSTEPPS®

Teamwork Perceptions Questionnaire

Instructions: Please complete the following questionnaire by placing a check mark [√] in the box that corresponds to your level of agreement from *Strongly Agree* to *Strongly Disagree*. Please answer every question, and select only one response for each question. The questionnaire is **anonymous**, so please do not put your name or any other identifying information on the questionnaire.

		Strongly Agree	Agree	Neutral	Disagree	Strongly Disagree
**Team Structure**						
1.	The skills of staff overlap sufficiently so that work can be shared when necessary.					
2.	Staff are held accountable for their actions.					
3.	Staff within my unit share information that enables timely decisionmaking by the direct patient care team.					
4.	My unit makes efficient use of resources (e.g., staff supplies, equipment, information).					
5.	Staff understand their roles and responsibilities.					
6.	My unit has clearly articulated goals.					
7.	My unit operates at a high level of efficiency.					
**Leadership**						
8.	My supervisor/manager considers staff input when making decisions about patient care.					
9.	My supervisor/manager provides opportunities to discuss the unit's performance after an event.					
10.	My supervisor/manager takes time to meet with staff to develop a plan for patient care.					
11.	My supervisor/manager ensures that adequate resources (e.g., staff, supplies, equipment, information) are available.					
12.	My supervisor/manager resolves conflicts successfully.					
13.	My supervisor/manager models appropriate team behavior.					
14.	My supervisor/manager ensures that staff are aware of any situations or changes that may affect patient care.					

PLEASE CONTINUE TO THE NEXT PAGE ⇨

		Strongly Agree	Agree	Neutral	Disagree	Strongly Disagree
**Situation Monitoring**						
15.	Staff effectively anticipate each other's needs.					
16.	Staff monitor each other's performance.					
17.	Staff exchange relevant information as it becomes available.					
18.	Staff continuously scan the environment for important information.					
19.	Staff share information regarding potential complications (e.g., patient changes, bed availability).					
20.	Staff meets to reevaluate patient care goals when aspects of the situation have changed.					
21.	Staff correct each other's mistakes to ensure that procedures are followed properly.					
**Mutual Support**						
22.	Staff assist fellow staff during high workload.					
23.	Staff request assistance from fellow staff when they feel overwhelmed.					
24.	Staff caution each other about potentially dangerous situations.					
25.	Feedback between staff is delivered in a way that promotes positive interactions and future change.					
26	Staff advocate for patients even when their opinion conflicts with that of a senior member of the unit.					
27.	When staff have a concern about patient safety, they challenge others until they are sure the concern has been heard.					
28.	Staff resolve their conflicts, even when the conflicts have become personal.					

PLEASE CONTINUE TO THE NEXT PAGE ⇨

		Strongly Agree	Agree	Neutral	Disagree	Strongly Disagree
**Communication**						
29.	Information regarding patient care is explained to patients and their families in lay terms.					
30.	Staff relay relevant information in a timely manner.					
31.	When communicating with patients, staff allow enough time for questions.					
32.	Staff use common terminology when communicating with each other.					
33.	Staff verbally verify information that they receive from one another.					
34.	Staff follow a standardized method of sharing information when handing off patients.					
35.	Staff seek information from all available sources.					
