# Supplementary material for: Interprofessional staff perspectives on the adoption of or black box technology and simulations to improve patient safety: a multi-methods survey
Source: Adv Simul (Lond). 2023 Oct 25;8:24. doi: 10.1186/s41077-023-00263-2 (PMC10598903; doi:10.1186/s41077-023-00263-2)
Supplement: Supplementary file 2 — Additional file 2. [file 41077_2023_263_MOESM2_ESM.pdf]

ORBB Simulation Perceptions Survey

Thank you for participating in this survey. As one of the first academic medical centers to adopt the powerful, emerging technology of Operating Rooms Black Box (ORBB) in Clements' and simulated ORs, we have an exciting opportunity to pioneer effective adoption of this technology to improve patient care.

Your feedback is highlighly valued. In order to effectively adopt this technology, it is critical to understand interprofessional OR teams' perspectives. The primary aim of this survey is to gain rich insights into team members' perspectives and buy-in around the adoption of ORBB into simulation opportunities at UTSW. Thus, this brief survey is being administered to OR stakeholders on campus, including surgeons, anesthesiologists, nurses, residents, technicians, and OR leaders. We hope that the investment of your time and inclusion of your ideas will help build a robust ORBB simulation program that promotes a safety culture, identifies areas for improvement, becomes an integral component for all team members, and leads to improved patient care. This survey has no bearing on your role or performance at UT Southwestern.

Participation in this survey is voluntary. All responses will be kept confidential and de-identified. If there are others on campus who you believe would be ideal in also providing insights on this topic, please let the survey administrator know, who is listed below.

We understand participation in this survey is a time commitment. This survey will take approximately 20 minutes to complete.

If you have questions or concerns when completing the survey, please reach out to Krystle Campbell, MS at Krystle.Campbell@utsouthwestern.edu.

Teamwork Perspectives on ORBB and Critical Event Simulations (AHRQ, 2021)

Instructions: This survey asks for your opinions about patient safety issues, errors, simulations, and event reporting. It will take about 15-25 minutes to complete. If a question does not apply to you or your hospital or you don't know the answer, please note the question "Does Not Apply or Don't Know."

Definitions:
• "Patient safety" is defined as the avoidance and prevention of patient injuries or adverse events resulting from the processes of healthcare delivery.
• A "patient safety event" is defined as any type of healthcare-related error, mistake, or incident, regardless of whether or not it results in patient harm.

1. What is your position in this hospital?	◯ Advanced Practice Nurse (NP, CRNA, CNS, CNM) ◯ Licensed Vocational Nurse (LVN), Licensed Practical Nurse (LPN)<br>◯ Hospital Aide, Nursing Assistant<br>◯ Registered Nurse (RN)<br>◯ Physician Assistant ◯ Resident<br>◯ Physician, Attending, Hospitalist<br>◯ Pharmacist, Pharmacy Technician<br>◯ Respiratory Therapist<br>◯ Technologist, Technician (e.g., EKG, Lab, Radiology) ◯ Supervisor, Manager, Department Manager, Clinical Leader, Administrator, Director<br>◯ Senior Leader, Executive, C-Suite<br>◯ Housekeeping, Environmental Services<br>◯ Information Technology, Health Information Services, Clinical Informatics<br>◯ Transporter ◯ Unit Clerk, Secretary, Receptionist, Office Staff<br>◯ Other, please specify:
	______________________________

2. Think of your "unit" as the work area, department, or clinical area of the hospital where you spend most of your work time. What is your primary unit or work area in this hospital?	◯ Many different hospital units, No specific unit ◯ Combined Medical/Surgical Unit<br>◯ Medical Unit (Non-Surgical)<br>◯ Surgical Unit ◯ Cardiology<br>◯ Emergency Department,<br>◯ Short Stay ◯ Gastroenterology<br>◯ ICU (all adult types) ◯ Labor & Delivery, Obstetrics & Gynecology<br>◯ Hematology ◯ Pediatrics (including NICU, PICU) ◯ Behavioral Health<br>◯ Pulmonology ◯ Physical Medicine<br>◯ Telemetry ◯ Surgical Services<br>◯ Anesthesiology ◯ Colonoscopy<br>◯ Pre Op, Operating Room/Suite, PACU/Post Op, Peri Op ◯ Lab ◯ Pharmacy<br>◯ Imaging ◯ Respiratory Therapy<br>◯ Social Services, Case Management, Discharge Planning ◯ Management<br>◯ Financial Services, Billing<br>◯ Human Resources, Training<br>◯ Information Technology, Health Information Management, Clinical Informatics<br>◯ Risk Management, Patient Safety Support Services ◯ Admitting/Registration<br>◯ Food Services, Dietary<br>◯ Environmental Services, Facilities<br>◯ Security Services ◯ Transport<br>◯ Other, please specify:
	__________________________________
3. How long have you worked in this hospital?	◯ Less than 1 year<br>◯ 1 to 5 years<br>◯ 6 to 10 years<br>◯ 11 or more years
4. In this hospital, how long have you worked in your current unit/work area?	◯ Less than 1 year<br>◯ 1 to 5 years<br>◯ 6 to 10 years<br>◯ 11 or more years
5. In your staff position, do you typically have direct interaction or contact with patients?	◯ I typically have direct interaction or contact with patients<br>◯ I typically do NOT have direct interaction or contact with patients
6. What is your age?	◯ Under 25 years old<br>◯ 26-35 years old<br>◯ 36-45 years old<br>◯ 46-55 years old<br>◯ 56-65 years old<br>◯ 66 + years old
7. Do you know what the OR Black Box is?	◯ Yes<br>◯ No

OR Black Box Technology - OR Black Box is a system that captures audio, visual, and other data on various aspects of surgery in order to help healthcare teams improve the quality and efficiency of patient care

8. Have you worked in an OR that has OR Black Box technology?

◯ Yes
◯ No

UT Southwestern OR Black Box and Simulation

1. How many team-based simulations have you participated in as a learner over the past 5 years?

◯ None
◯ 1 to 2
◯ 3 to 5
◯ 6 to 10
◯ 11 or more

2. What benefits, if any, do you believe the OR Black Boxes provide?

____

3. What concerns, if any, do you have with black boxes being integrated?

____

4. What benefits, if any, do you believe integrating OR Black Box technology with simulation opportunities will provide?

____

5. What concerns, if any, do you have with integrating OR Black Boxes technology with simulation opportunities?

____

6. What would enable effective adoption of OR Black Box-enhanced simulations?

____

7. What barriers are present that might impact the adoption of OR Black Box-enhanced simulations?

____

8. Please share any additional feedback that you believe is important for the OR Black Box project.

____
